# The alteration of brain function in overweight/obese individuals and the neurological benefit of thymoquinone: uncovering molecular mechanisms

**DOI:** 10.1007/s11011-026-01826-8

**Published:** 2026-04-01

**Authors:** Mostafa D. Mostafa, Magda A. El-Komy, Maggie E. Amer, Azza I. Othman, Mohamed A. El-Missiry

**Affiliations:** Zoology Department, Faculty of Science, Mansoura University, Mansoura, Egypt

**Keywords:** Brain cognitive function, Obesity, High-fat diet, Thymoquinone, Oxidative stress, Inflammation

## Abstract

Overweight and obesity are complicated metabolic disorders associated with negative impacts on the brain and its function, including cognitive and memory abilities. Herbal medicines are plant-based bioactive compounds produced by plants. Natural food components with antioxidant activity show promise as alternative treatments for a number of illnesses, including brain diseases. Thymoquinone (TQ) is the main bioactive constituent of essential oils produced from *Nigella sativa* seeds. TQ possesses numerous biological and pharmacological activities, including antioxidant, anti-inflammatory, antihyperglycemic, and immunomodulatory effects, with neuroprotective effects against various neurodegenerative diseases and pathological conditions of the brain. Several studies have investigated the pharmacological activities of TQ; however, its neuroprotective molecular signaling pathways have not been fully described, and several issues remain to be clarified. The current review summarizes the most recent information related to the important molecular signaling pathways by which TQ protects brain function, particularly in overweight/obese individuals. In conclusion, TQ improved the activity of antioxidant enzymes, maintained redox equilibrium and decreased neuroinflammation and apoptosis via various signaling pathways. Numerous proteins, including nuclear factor erythroid 2–related factor 2 (Nrf2), nuclear factor kappa B (NF-κB), toll-like receptor 4 (TLR4), and mitogen-activated protein kinase (MAPK), are crucial for the TQ signaling pathway to prevent brain problems. Although these proteins represent a novel treatment approach, there are still issues with their clinical applicability in various diseases. Researchers should perform further research to determine the additional activity of TQ for the development of clear strategies for the prevention and treatment of brain dysfunction.

## Introduction

Learning and memory are important cognitive functions of the human brain and are identified as the capacity of the brain to recognize, save and recover information. Worldwide, cognitive decline and brain injury are the most serious health problems linked to obesity in young, adult and elderly people. Numerous factors contribute to brain dysfunction in obese and overweight people, starting with poor lifestyles and dietary habits, genetics, the environment, and underlying medical conditions (Rukadikar et al. 2023).

Overweight and obesity are characterized by increased chronic inflammation, initiated by hypertrophied adipose tissue which release pro-inflammatory cytokines and adipokines, such as tumor necrosis factor-alpha (TNF-α), interleukin-6 (IL-6), and leptin. This systemic inflammatory state leads to elevated oxidative stress, mitochondrial dysfunction, insulin resistance, and reduced autophagy in all organs, particularly the nervous system (Naomi et al. 2023). The systemic inflammatory mediators can affect the integrity of the blood–brain barrier (BBB), which basically protects the brain from peripheral insults. Chronic inflammation disrupts tight junction proteins and promotes a pro-inflammation in BBB cells, by increasing its permeability (S. Kim et al. 2025). The disruption in BBB acts as a critical link between peripheral obesity and central nervous system pathology. This triggers neuroinflammation, increased oxidative stress, and directly impairs synaptic plasticity, neuronal function, and cognitive functions as well (Tucsek et al. 2014). It is suggested that appropriate antioxidants that modulate the biological processes linked to obesity might mitigate neurological dysfunction, hence restoring cognitive functions. Natural bioactive molecules derived from plants have been shown to have a wide range of biological effects, including antioxidant effects that reduce oxidative damage, moderate inflammation, control autophagy, and inhibit apoptosis to maintain cell survival (Sura and Cheng 2024; Yu et al. 2022; Elkatry et al. 2022; Ekiert and Szopa 2020; Anwar et al. 2020). Thymoquinone (TQ) is the main bioactive substance of the essential oil of black cumin (*Nigella sativa*) seeds. TQ has shown remarkable therapeutic efficacy against several diseases, including diabetes and neurodegenerative disorders (Hafez et al. 2024).

Currently, our understanding of how TQ signaling pathways counteract the negative effects of overweight and obesity on brain function and cognitive decline is lacking. The purpose of the present review is to highlight recent signaling mechanisms through which TQ exerts biological and pharmacological benefits, preventing the cascade from systemic inflammation and BBB disruption to neurocognitive decline associated with obesity.

## Methods

Reviews and experimental studies were selected on the basis of their relevance to the objectives of this review and the searches conducted on PubMed/MEDLINE and ScienceDirect. Among the search parameters, the phrases thymoquinone, obesity, overweight, neurodegeneration, brain disease, neuroinflammation, oxidative stress, mitochondrial function, and autophagy were combined.

## Prevalence of obesity/overweight

High-fat and caloric foods are found in the western diet and fast-food diet, which are common in meals of workplaces, restaurants, and schools. Most foods that are fried or cooked with excess oil are considered high-fat foods. Generally, a high-calorie and high-fat diet encounters the following criteria, including consuming more calories than the body needs, fat constituting 30%−60% of energy intake, and the presence of high saturated and trans fats in the meal (Balakrishnan et al. 2024; Chooi et al. 2019).

Both neurodevelopmental disorders and neurodegenerative diseases are linked to obesity, which may be associated with dietary habits and environmental and genetic factors (Flores-Dorantes et al. 2020; Loos and Yeo, 2022). An energy imbalance caused by excessive consumption of high-energy food and insufficient exercise is the main cause of overweight and obesity (Feng et al. 2024). For the past 50 years, the prevalence of overweight and obesity has markedly increased globally, accounting for half of the global population (Bora and Fisette 2023). Regardless of geographic location, ethnicity, or economic status, obesity rates have increased across all ages and sexes; nevertheless, older people of both genders and women of all ages are more prevalent (Chooi et al. 2019). In all cases, the main cause of excessive weight gain and obesity is sedentary behavior, which is encouraged by technological advancements and the increased availability of highly processed foods high in fats and/or sugar (Wong Zhang et al. 2023).

## Overweight/obesity and cognitive impairment

Overweight/obesity are complicated metabolic disorders and are recognized as excessive fat accumulation, which is considered a health risk factor (Harborg et al. 2024). Overweight and obesity are associated with various physiological alterations, including increased inflammation, insulin resistance, oxidative stress and impaired autophagy, leading to structural and biological brain changes (Gómez-Apo et al. 2021; Behrooz et al. 2024). A recent study investigated the effects of caloric diet diets on anxiety, learning, and memory as well as hippocampal neurogenesis and neuroinflammation in aged rats (Mota et al. 2023a, b). They concluded that increased consumption of fat and sugar has been linked to deficits in hippocampus-dependent learning and memory in both adults and children, indicating a detrimental effect on hippocampal function throughout life (Schmitt and Gaspar 2023).

Memory impairment appears to be linked to diet-induced hypercholesterolemia. After two months of being fed a diet high in cholesterol (1.25% cholesterol and 20% fat), Swiss mice showed signs of short-term memory impairment. Furthermore, rats exposed to a high-cholesterol diet developed substantial deficiencies in long-term memory and spatial learning (Posridee et al. 2023). Recent findings demonstrated that consuming deep-fried and reheated oil on a regular basis increased the levels of serum triglycerides and oxidative stress markers (Balakrishnan et al. 2024). Studies using magnetic resonance imaging have shown that obese patients exhibit regional brain atrophy as well as abnormalities in gray and white matter, indicating a link between obesity and cognitive impairment (Chen et al. 2022a, b).

Childhood obesity increases the possibility of cognitive impairment, which has become a serious global health concern among school-aged students. Compared with healthy adolescents, obese students have worse cognitive function levels, especially attention, retention, intelligence, and cognitive flexibility (Meo et al. 2019). Compared with normal individuals, overweight/obese children and adolescents have been shown to have less cognitive function on verbal, full-scale, and performance IQ; visual–spatial, and executive function tests (Snyder et al. 2022). Early to midlife, obesity is associated with an earlier start of cognitive impairment in a variety of ways. However, lifestyle interventions to encourage weight loss can reverse cognitive dysfunction, especially dieting (Costache et al. 2023).

Obese patients have a high risk of Alzheimer's disease (AD). Studies have revealed that low 42/40 ratios of plasma amyloid-β (Aβ), which are indicative of dyshomeostasis, are linked to an increased risk of AD and that Aβ40 appears to decrease with weight loss. This finding suggests that body mass index and weight loss in overweight and obese individuals are associated with plasma Aβ homeostasis (Brook et al. 2023). The authors of a recently published study demonstrated that miRNAs can be transferred from adipose tissue to the hippocampal region via extracellular vesicles and are markedly increased in individuals with diabetes and obesity. Among the increased miRNAs, miR-9-3p was consistently upregulated in the extracellular vesicles of diabetic patients and the hippocampal tissues of mice fed a high-fat diet (HFD), causing both synaptic injury and cognitive impairment (J. Wang et al. 2022). Furthermore, excessive HFD consumption exacerbates glutamate-mediated excitotoxicity, leading to impairment of synaptic plasticity (Mostafa et al. 2025).

Numerous studies have described the putative mechanisms that obesity and neurodegeneration share; these mechanisms are interconnected and impact/stimulate other pathways. These mechanisms are potential targets for mitigating obesity-induced brain dysfunction. A summary of the effects of high-calorie diets on cognitive dysfunction in experimental animal models is provided in Table 1.

**Table 1:** Effects of high-calorie diets on cognitive dysfunction in experimental animal models

Strain	Age	Diet	Feeding treatment	Outcome	References
Male C57BL/6 J mice	13 months	60% kcal fat	24 weeks	• Progressive change in the cognitive function of the hippocampus • Increased expression of Aβ in the cornu ammonis 3 (CA3) of the hippocampi	(Chen et al. 2022a, b)
Female Wistar albino rats	4 months	20% fructose	11 weeks	• Tau hyperphosphorylation in the hippocampus • Increased interleukin-1β (IL-1β) levels in the CA1 region of the hippocampus	(Pérez-Corredor et al. 2022)
Male Wistar rats	18 months	20.1% fat and 14% sugar	12 weeks	• Impairment of spatial learning and working memory • Increase in neuroinflammation in the hippocampus	(Mota et al. 2023a, b)
Male Wistar rats	8 weeks	20% carbohydrates and 60% fat	12 weeks	• Mitochondrial dysfunction in the hippocampus and cortex • Deficit in hippocampal- mediated spatial memory	(Vilela et al. 2024)
Swiss male mice	2 months	26% carbohydrates and 59% lipids (oil and lard)	16 weeks	• Impairment of cognitive function, increase in neuronal apoptosis in the cortex • An increase in protein oxidation and a decrease in superoxide dismutase (SOD) and catalase (CAT) activity in the cortex	(Luciano et al. 2024)
C57BL/6 J male mice	11 weeks	55% kcal fat	15 weeks	• Disruption in synaptic plasticity was associated with elevated levels of pro-inflammatory cytokines in the hippocampus, including IL-1β, interleukin-6 (IL-6), and tumor necrosis factor-alpha (TNF-α)	(Shi et al. 2020)
Male C57BL/6 J mice	5 Weeks	43% carbohydrates and 42% fat	16 weeks	• Impairment of long-term potentiation in the dentate gyrus • Increased levels of TNF-α, IL-6, and IL-1β and lipid peroxidation in the hippocampus and cortex • Disruption in synaptic plasticity	(Liu et al. 2014)

Recently, researchers showed several brain complications derived from obesity. While the exact mechanisms linking obesity to neurodegeneration remain unclear, it is believed that chronic inflammation, insulin resistance, oxidative stress, and BBB disruption are shared features that further exacerbate CNS diseases. More studies are needed to address the complex interplay between obesity and neurodegeneration to develop effective prevention and treatment approaches to prevent neurological diseases progression, focusing on weight loss and healthier diets.

## Role of oxidative stress in high-fat diet-induced neurodegeneration

An imbalance between antioxidant levels and reactive oxygen species (ROS) generation, known as oxidative stress, leads to the disruption of redox signaling in all cells (Sies 2020). One of the main causes of the onset and progression of cognitive impairment in metabolic disorders is oxidative stress. The high amount of polyunsaturated fatty acids and high oxygen consumption make the brain extremely vulnerable to oxidative damage (Wen et al. 2023). There are close associations between oxidative stress, insulin resistance, neuroinflammation, dyslipidemia and cognitive impairment. Furthermore, consuming a HFD accelerates the mitochondrial β-oxidation of free fatty acids, which increases the production of ROS (Aranda-Rivera et al. 2022). Obesity has been linked to defects in mitochondrial biogenesis in the hypothalamus. Recent research has shown that high-calorie diets cause obesity in female rats and significantly reduce the levels of antioxidant enzymes in the hypothalamus, including CAT, glutathione reductase (GR), and SOD (Kovačević et al. 2019). Adipocyte hypertrophy mediates mitochondrial dysfunction and endoplasmic reticulum stress and promotes the activation of NADPH oxidase, nitric oxide synthase (NOS) and uncoupled endothelial NOS. These events stimulate excessive generation of ROS, which results in accelerated synthesis of proinflammatory cytokines and nuclear factor kappa B (NF-κB) by increasing the production of adipokines (Masenga et al. 2023).

Nuclear factor erythroid 2-related factor 2 (Nrf2) is the upstream gene expression protein of the antioxidant signaling pathway and the primary regulator of oxidative‒redox equilibrium (Abdalkader et al. 2018). Nrf2 malfunction increases the negative effects of obesity, including cerebrovascular and brain health, by impairing neurovascular coupling mechanisms, blood–brain barrier (BBB) integrity and synaptic function and promoting neuroinflammation (Tarantini et al. 2018; Xia et al. 2022).

Nrf2 deficiency leads to an increase in the levels of Aβ in AD model mice and AD patients. Conversely, the upregulation of Nrf2 decreases the production of beta-secretase beta-site APP cleaving enzyme 1 (BACE1) and facilitates the autophagy-mediated degradation of phosphorylated tau, which further improves cognitive function (Bahn et al. 2019; Jo et al. 2014). A recent report showed that Nrf2 promotes macroautophagy and chaperone-mediated autophagy to eliminate tau and amyloid precursor proteins by preserving proteostasis (Qu et al. 2020). A recent study revealed that the Keap1/Nrf2 pathway is intimately related to various pathways involved in the development of neurodegenerative diseases (He and Sun 2021). Nrf2 activation can enhance mitochondrial bioenergetics, which may help neurons comply with their high energy requirements. Moreover, to prevent neuroinflammation, Nrf2 suppresses the NLRP3 inflammasome, restores cytokine release to normal levels and prevents NF-κB (He and Sun 2021).

Nrf2 is a transcription factor that regulates the cellular antioxidant capacity and decreases oxidative stress. It regulates the transcription of numerous ROS-detoxifying enzymes, including glutathione peroxidase (GPx), heme oxygenase-1 (HO-1), glutathione (GSH) and thioredoxin (TrX) (Tonelli et al. 2018; He et al. 2020). Interestingly, the Nrf2 signaling pathway is inhibited by NF-κB by increasing the activity of Kelch-like ECH-associated protein 1 (Keap1), which results in Nrf2 ubiquitination and decreased Nrf2 binding to its associated DNA regions (Gao et al. 2022). According to Zang et al., consuming a HFD can cause overproduction of ROS, high levels of malondialdehyde (MDA), and low levels of GSH, decrease SOD activity and impair Nrf2 and Nrf2-regulated enzyme expression, which may lead to impaired learning and memory in the cerebral cortex of mice (Zhang et al. 2021).

**Fig. 1:**
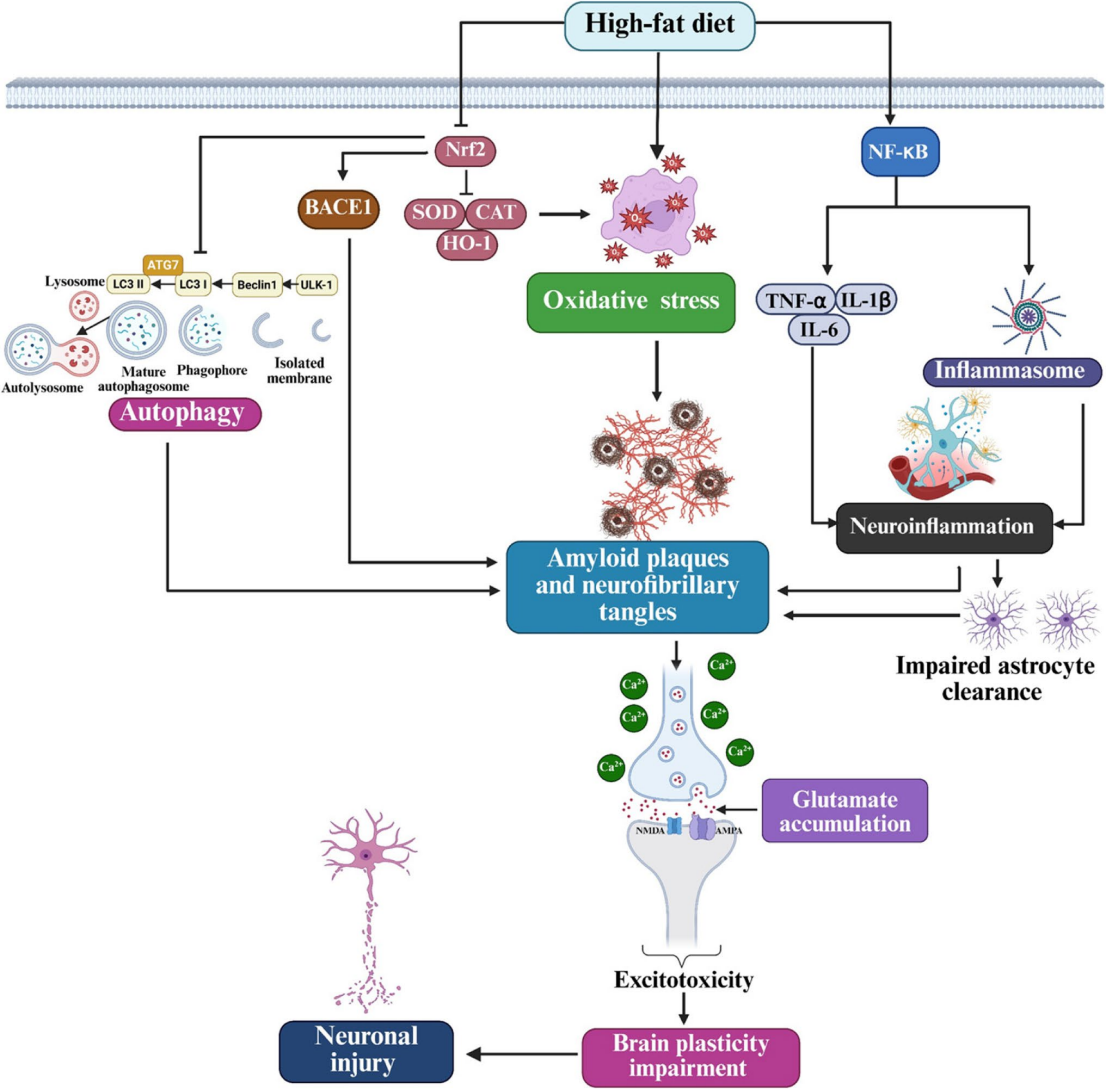
HFD impairs brain plasticity via Nrf2 suppression and NF-κB activation: a pathway of oxidative Stress, neuroinflammation, and autophagy dysfunction. Excessive consumption of a HFD causes oxidative stress by down-regulating Nrf2 signaling pathway with subsequent decrease in the expression of antioxidant enzymes (SOD, CAT, and HO-1). Dysregulation of Nrf2 by HFD consumption leads to BACE1 activation which help in the synthesis of amyloid-β peptide. Additionally, HFD has been linked to impaired autophagy through ATG7, LC3, Beclin1, and ULK-1 pathway. In parallel with these events, HFD activates NF-κB inflammatory pathway which stimulates the release of TNF-α, IL-1β, IL-6, and inflammasome activation, contributing to neuroinflammation, oxidative stress and impaired astrocyte-mediated clearance. These pathological changes facilitate the accumulation of amyloid plaques and neurofibrillary tangles, glutamate accumulation, and Ca^2^⁺ dysregulation, resulting in excitotoxicity, impaired brain plasticity, and neuronal injury. ATG-7: autophagy related 7; BACE1: beta-site APP cleaving enzyme 1; Beclin-1: coiled-coil myosin-like BCL2-interacting protein; CAT: catalase; HO-1: heme oxygenase-1; IL-1β: interleukin-1 beta; IL-6: interleukin-6; LC3-I: microtubule-associated protein 1 light chain 3-I; LC3-II: microtubule-associated protein 1 light chain 3-II; NF-κB: nuclear factor kappa B; Nrf2: nuclear factor erythroid 2–related factor 2; SOD: superoxide dismutase; TNF-α: tumor necrosis factor alpha; ULK1: unc-51-like kinase 1

According to the above summary (Fig. 1), oxidative stress is a major factor in neurocognitive dysfunction and increases the susceptibility of the central nervous system to damage caused by the downregulation of antioxidants and excessive production of ROS. Therefore, oxidative stress is considered a cornerstone therapeutic target, and adequate antioxidant action can decrease the oxidative damage that results from ROS due to the overconsumption of a high-caloric diet. It is believed that antioxidant agents have the ability to target oxidative stress by eliminating ROS in cases of obesity-induced neurocognitive dysfunction.

## Role of mitochondrial dysfunction in HFD-induced cognitive impairment

Neuronal mitochondria are essential for maintaining axonal structure, supplying energy for neurotransmitter recycling, ion channel and receptor function, and promoting local protein synthesis, which is required for synaptic plasticity (Song et al. 2025). Normally, mitochondria protect neurons by reducing oxidative damage and sustain neuronal activity by supplying neurons with an adequate amount of energy. Numerous studies have indicated that impaired mitochondria play a major role in the etiology of brain diseases, particularly in terms of cognitive and memory functions (Wang et al. 2020). Recently, pathological tau formation has been associated with mitochondrial malfunction, cognitive decline, and synaptic failure in neurological disorders and aging (Olesen et al. 2024).

Owing to its high energy needs and susceptibility to oxidative stress, mitochondrial dysfunction is especially prevalent in the brain. Consequently, mitochondrial dysfunction and oxidative stress can cause a range of neuropsychiatric and neurodegenerative conditions (Bhatt et al. 2020). The main relationship between neurodegeneration and mitochondrial dysfunction involves ROS. HFD intake impairs respiratory chain complexes and collapses the mitochondrial membrane potential (Chen et al. 2018). Furthermore, the brain cortex of mice fed 50% fat for 18 weeks presented increased levels of mitochondrial hydrogen peroxide (H_2_O_2_) and superoxide anion (O_2_^•–^) production and decreased levels of antioxidant enzymes (Cavaliere et al. 2019a, b).

Recent research has shown that a long-term HFD impairs mitochondrial brain function and increases insulin resistance, inflammation and cellular oxidative damage in the brain (Schmitt and Gaspar 2023), indicating that mitochondrial dysfunction is associated with brain malfunction and cognitive deficits. Earlier findings also revealed that HFD-fed rats exhibit obesity-induced brain mitochondrial depolarization and swelling as well as increased oxidative stress in the brain and hippocampus, decreased hippocampal plasticity, and increased apoptosis, which contribute to cognitive decline (Sa-Nguanmoo et al. 2018).

Dysfunctional synaptic mitochondria may be associated with impaired neurotransmission. The maintenance of cellular Ca^2+^ homeostasis is largely dependent upon mitochondria. On the other hand, the inner mitochondrial membrane in the brains of obese animal models becomes more permeable to ions and solutes because of an excess of Ca^2+^ within the mitochondria. These changes contribute to the generation of ROS, a reduction in ATP synthesis, the opening of the mitochondrial permeability transition pore, the release of cytochrome c, and ultimately the triggering of neuronal apoptosis and cognitive impairment (Ahmed et al. 2021; Clemente-Suárez et al. 2023; Tian et al. 2023).

According to these studies, targeting metabolic and energetic abnormalities in mitochondria might effectively protect against and restore brain plasticity in neurological and cognitive diseases caused by obesity.

## Role of inflammation in obesity-induced neurodegeneration

Neuroinflammation is a characteristic risk factor for neurodegeneration that decreases synaptic plasticity and motor and cognitive functions. Cavaliere et al. reported that 18-week HFD-fed male C57BL/6 J mice presented significant increases in the levels of IL-6, IL-1β, TNF-α and leptin in the brain cortex (Cavaliere et al. 2019a, b). Additionally, adipose tissue hypertrophy is correlated with increased expression of proinflammatory cytokines (Kirichenko et al. 2022). Interestingly, systemic low-grade chronic inflammation linked to HFD-induced obesity also disrupts BBB permeability. Consequently, the brain is more prone to toxins and proinflammatory cytokines that activate microglia (Salas-Venegas et al. 2022). Activated microglia stimulate the activation of NF-κB and activator protein 1 (AP-1), which in turn increases the expression of proinflammatory cytokines and chemokines. Furthermore, Wang and colleagues demonstrated that 18 weeks of HFD consumption in male C57BL/6 J mice triggers hippocampal neuroinflammation and dysfunction by increasing the expression of the mRNAs IL-1β, IL-6, and TNF-α (S. Wang et al. 2023). Furthermore, proinflammatory cytokines are stimulated by obesity-induced hyperleptinemia. NF-κB is significantly activated by leptin, which stimulates proinflammatory genes that are linked to inflammation (Czaja-Stolc et al. 2022). Inflammation contributes to insulin resistance, which is associated with neurocognitive dysfunction (Stoeckel et al. 2016). A recent study demonstrated that long-term consumption of hypercaloric diets causes oxidative stress and a proinflammatory state in brain regions, including the frontal cortex, hippocampus, and hypothalamus. This can result in dysfunction, altered metabolism, neuronal damage, and loss of recognition memory (Fuentes et al. 2023).

The consumption of fatty acids stimulates the innate immune system via Toll-like receptors (TLRs), which in turn trigger the inflammatory response and the activation of immune cells (Howe et al. 2022). During the development of obesity, immune cells, primarily macrophages, infiltrate the expanding white adipose tissue. Increased expression of proinflammatory cytokines, including IL-6, IL-1β, and TNF-α, is also linked to the development of adipose tissue. The development of neuroinflammation might affect the BBB. It has been reported that increased BBB permeability and neuroinflammation trigger reduced hippocampal function in short-term HFD-fed mice (de Paula et al. 2021). The behavioral and BBB abnormalities induced by HFD-feeding diminished in mice receiving a TNF-α inhibitor, indicating that inflammatory signaling is an essential contributor to these modifications (de Paula et al. 2021). According to Jin et al., excess free fatty acids that accumulate as ectopic fats lead to increased expression of proinflammatory cytokines and ROS, which in turn induce neurotoxicity, cognitive impairment, motor impairment, and BBB failure (Jin et al. 2023).

Inflammation is a prominent pathological event observed in neurodegenerative diseases and cognitive impairment; therefore, it could be a useful therapeutic target in the treatment of brain injuries and neurodegeneration. Consequently, anti-inflammatory agents have been suggested as potential approaches for reducing the risk or preventing the onset of neuroinflammation, hence improving neurocognitive performance.

## Role of autophagy in HFD-mediated neurodegeneration

Autophagy is an intracellular, lysosome-dependent mechanism for degrading misfolded proteins and damaged organelles. This process can be initiated by the formation of an autophagosome, a vesicular double-membrane structure, which engulfs misfolded and aggregated proteins and fuses with lysosomes for further degradation. Autophagy efficiency is suppressed in obesity, insulin resistance, intracellular oxidative stress and inflammation (Udoh et al. 2022). The levels of the autophagy-related proteins Atg5, Atg5-Atg12, and LC3-II are decreased, whereas the level of the p62 protein is increased in the livers of HFD-fed mice (Korovila et al. 2021). Under physiological conditions, autophagy can be regulated through the mTOR/AMPK signaling pathway (Rabanal-Ruiz et al. 2017).

Li and coworkers reported that HFD consumption suppressed autophagy in the hippocampus of HFD-fed mice by increasing the p-mTOR/mTOR ratio and decreasing the p-AMPK/AMPK ratio. Furthermore, the hippocampal levels of p62 are elevated, whereas the levels of Atg7, Beclin1, and LC3 are decreased in the same animals (Li et al. 2022). However, treatment with rapamycin (an mTOR signaling pathway inhibitor) ameliorated the impaired autophagy caused by HFD consumption. Moreover, reduced autophagic activity (marked by downregulation of Beclin1 and upregulation of p62) is associated with a decrease in spatial memory and cognitive function in aged diabetic mice (Guan et al. 2016).

Sirtuin 1 (SIRT1) is an NAD^+^-dependent deacetylase that regulates autophagy, inflammation and apoptosis (Yang et al. 2022). A HFD disrupts autophagy by inhibiting the SIRT1/AMPK pathway (Mei et al. 2022). AMPK promotes the activation of SIRT1, which in turn deacetylates autophagy-related proteins (Han et al. 2023). Moreover, high fat intake impairs autophagy, synaptic plasticity, and cognitive function by downregulating SIRT1/AMPK and lowering the levels of LC3, Atg7, Atg12, Beclin1, and Atg12 in the hippocampus (Yi et al. 2024).

The current overview of the relationship between brain function and autophagy may offer new therapeutic options for obesity-induced neurological disorders, thereby helping patients with neurocognitive impairment.

## Plant-based natural products:

Natural products are the main sources of pharmaceutical drugs, and the plant kingdom is a rich source of bioactive compounds (Gruber 2024; Wiyarta et al. 2025). These natural compounds have various biological and physiological activities, such as antioxidant, anti-inflammatory, and antiapoptotic activities, and modulate autophagy (Sura and Cheng 2024; Yu et al. 2022; Elkatry et al. 2022; Ekiert and Szopa 2020; Anwar et al. 2020). Natural bioactive compounds have a wide range of therapeutic benefits and could be used as medicinal, adjuvant or preventative agents to improve brain health (Alza et al. 2024). A plethora of dietary supplements, herbal therapies, and medical foods are being promoted as beneficial neuroprotective supplements and memory enhancers to prevent or delay neurocognitive impairment (Reza-Zaldivar and Jacobo-Velazquez 2023). In recent years, secondary metabolites such as polyphenols, alkaloids and flavonoids have been shown to exert protective effects against cancer, cardiovascular diseases, obesity and neurodegenerative diseases (Abdallah et al. 2023). Owing to a number of factors, including fewer adverse effects, accessibility, and ease of administration, herbal treatment has been widely used to treat a variety of neurological conditions (Salm et al. 2023). Bioactive nutritional molecules are unique because of their wide range of antioxidant and anti-inflammatory functions. Furthermore, functional dietary components can protect against and repair DNA damage and modulate signal transduction pathways and gene expression in cells (Nosrati et al. 2017).

### Phytochemical profile of *Nigella sativa*

*Nigella sativa* L. (black seed) is well known to have a broad therapeutic potential attributed to its rich phytochemical composition. More detailed profile is required for standardization and quality control in pharmacological research. The seeds contain a diverse array of bioactive constituents, which can be classified into volatile and non-volatile fractions, where their relative abundance influenced by genetic factors, geographical origin, cultivation practices, and post-harvest processing (Arshad et al. 2025).

- **Volatile (essential) oil (≈0.4–1.6% w/w of seeds):** The most pharmacologically significant fraction (Botnick et al. 2012). **Benzoquinones:** Thymoquinone (TQ) is the main pharmacologically bioactive constituent, representing 15–40% of the seed essential oil with reported values from ~ 1.6–42% depending on origin and preparation method. Other related quinones include thymohydroquinone (THQ), dithymoquinone (nigellone), and thymol (Degu et al. 2025).
- **Monoterpene hydrocarbons (≈80–90% of volatiles):** basically p‑cymene (roughly 20–40% of the essential oil, a biosynthetic precursor for TQ, in addition to γ‑terpinene, α‑thujene/β‑thujene, α‑pinene, and β‑pinene (Arshad et al. 2025).
- **Fixed oil (≈30–45% w/w of seeds):** rich in unsaturated fatty acids (linoleic acid, ~ 45–58%); oleic acid, ~ 20–25%), phytosterols (e.g., β-sitosterol), and tocopherols (Vitamin E).
- **Other bioactive constituents:** Includes triterpene, saponin α-hederin, various isoquinoline alkaloids (e.g., nigellicine, nigellimine), flavonoids, and proteins (M. Abbas et al. 2024).

### Thymoquinone

The Nigella sativa seed was used from 3000 years ago by ancient Assyrian and Egyptian civilizations for dietary, cosmetic and remedy purposes. Several containers of Nigella sativa oil were found in the tomb of the Egyptian pharaoh, King Tutankhamun (Padhye et al. 2008). Thymoquinone (TQ) was first isolated as the principal bioactive component from black seeds in 1963 by El-Dakhakhny (El–Dakhakhny 1963). Thymoquinone (TQ; 2isopropyl5-methyl-1,4benzoquinone) is the natural phytochemical and active ingredient of the essential oil obtained from *Nigella sativa* seeds. It is a lipophilic monoterpene diketone with a molecular weight of 164.204 g/mol. Owing to its low molecular weight and lipophilic nature, which allow it to pass through biological membranes such as the BBB, it is believed to have potential therapeutic benefits for the nervous system (Sarkar et al. 2021). The occurrence and bioactivity diversity of TQ was reviewed previously (Samra et al. 2022). TQ shows high potential for drug development because of its pharmacological characteristics, pharmacokinetics, efficacy, high therapeutic index, lipophilicity, and safety margin. The pharmacological properties of TQ include antioxidant, anti-inflammatory, immunomodulatory, antimicrobial, and anticancer. The pharmacokinetics & stability of TQ are attributed to its lipophilicity, which increases its absorption, protein binding affinity to plasma protein, great stability in acidic medium, and high safety margin (ElKhoely et al. 2015).

#### Analytical techniques for thymoquinone detection and quantification

Choosing the extraction method directly depends on TQ yield and stability. Solvents like *n*-hexane yield high TQ content, whereas aqueous/ethanol extracts may contain little TQ despite high total yield (Iqbal et al. 2018). Accelerated solvent extraction (ASE) allow rapid, high-yield extraction/quantification (R. Ahmad et al. 2020).

#### Chromatographic separation and detection

- **Gas chromatography-mass spectrometry (GC–MS):** Usually used for TQ in oils; it allows selective, validated quantification and product comparison via characteristic mass fragmentation (Tekbaş et al. 2023).
- **High-performance liquid chromatography (HPLC/UHPLC):** This is the primary method for the direct quantification of TQ in complex matrices without the need for volatilization (Dedić et al. 2024).
- **High-performance thin-layer chromatography (HPTLC):** Multiple validated and cost-effective methods use normal phase plates and n-hexane/ethyl acetate or similar systems, scanning at 254–259 nm for routine QC of extracts, oils and formulations (Dedić et al. 2024).

#### Electrochemical & UV methods

Square-wave voltammetry with carbon paste electrodes that provides a low-cost, sensitive alternative method for oils and supplements, with strong agreement versus HPLC (Świderski et al. 2025). Method Validation and Stability for all quantitative assays, validation according to International Council for Harmonisation (ICH) guidelines is essential, covering specificity, linearity, precision, accuracy, robustness, and sensitivity (Tekbaş et al. 2023; Dedić et al. 2024; Świderski et al. 2025).

#### Synthesis and production of thymoquinone

TQ is regularly prepared by the sulfonation/oxidation of thymol or carvacrol (Darakhshan et al. 2015; Aftab et al. 2020; Ahmad et al. 2019).

The low natural yield and instability of TQ have directed the research into synthetic and biotechnological production that remarkably enhance the selectivity and yield of TQ.

- Homogeneous Mn(II)–bipyridine catalyst (Mn(II)/TBHP/MeCN) converts thymol and carvacrol to TQ with 100% selectivity (Kani et al. 2024).
- A water‑soluble Mn(III) PEG–porphyrin in a biphasic water/hexane system oxidizes thymol and carvacrol with 78–94% conversion and allows simple catalyst recycling; oregano oil thymol/carvacrol can be selectively transformed to TQ (Neves et al. 2018).These techniques use cheap monoterpenes and show high yields, but requires controlling oxidant cost, solvent management, catalyst lifetime, and overall economics versus optimized extraction (Kani et al. 2024).

#### Thymoquinone role in mitigating brain injury

TQ has been shown in recent experiments to effectively improve neurological deficiency scores, reduce cerebral infarction, and improve the morphology and number of cells in infarcted areas (Fan et al. 2020). Through a variety of interconnected biological processes, such as the regulation of redox balance, the inflammatory response, neurotransmitter equilibrium, and the regression of DNA damage and apoptosis, TQ improved body weight gain and metabolic status in obese rats while attenuating brain injury, cognitive impairment, and memory deficit (Mostafa et al. 2025). In addition, several clinical studies have explored the clinical utility of TQ in various human conditions, including metabolic syndrome (diabetes mellitus and obesity), cancer, COVID-19, epilepsy and chronic periodontitis (Gouda et al. 2025).

TQ has a broad range of activities, including neuroprotective activity in models of brain diseases such as traumatic brain damage, Alzheimer's disease, Parkinson's disease, and brain ischemia/reperfusion (Isaev et al. 2020). The neuroprotective effect of TQ is attributed to its antioxidant properties. Regarding the pathophysiology of brain injury, the primary goals of treatment should be to suppress inflammatory processes and reduce the overproduction of ROS. *In both in vivo* and *in vitro* experiments, TQ activated the Nrf2 and OH-1 signaling pathways to reduce inflammation and oxidative stress and prevent apoptosis and autophagy in a cerebral ischemia model of brain injury. These findings indicate that the effect of TQ is mediated by a reduction in oxidative stress and neuronal apoptosis (Amin et al. 2021).

Numerous investigations have been carried out in an attempt to clarify the molecular signaling pathways and mechanisms that underlie the various pharmacological characteristics of TQ. The aim of the next section is to identify the neuroprotective and mitigating effects of TQ and to identify the main mechanisms and signaling pathways involved in improving brain health (Table 2).

**Table 2:** Preclinical trials for assessing the neuroprotective effects of various doses and routes of administration of thymoquinone (TQ) on brain injury

Strain	Dose	Dose route	Effects	Reference
Male Sprague‒Dawley rats	40 mg/kg/day for 5 days	Oral gavage	TQ reduces NADPH oxidase-2, NADPH oxidase-4, MDA levels, and increase CAT and SOD activity	(Bilgiç et al. 2023)
Male Wistar albino rats	5 mg/kg/day for 7 days	Intraperitoneal injection	Decrease in MDA and increase in SOD and CAT levels in rat brain	(Ceylan et al. 2023)
Male Sprague‒Dawley rats	10 mg/kg twice	Intraperitoneal injection	Increase the expression of Nrf2, HO-1 and SOD in the hippocampus	(Shao et al. 2017)
Male Wistar albino rats	10 mg/kg/day for 4 weeks	Oral gavage	Upregulation of total antioxidant capacity in hippocampus	(Akcay et al. 2024)
Male albino Wistar rats	5 and 10 mg/kg/day for 56 days	Intraperitoneal injection	Decrease MDA levels and enhance GSH concentration in hippocampus	(Oskouei et al. 2021)
Male albino Wistar rats	2.5 and mg/kg/day for 3 days	Oral gavage	Reduce oxidative stress and DNA damage in the hippocampus	(Firdaus et al. 2018)
Male albino Wistar rats	10 mg/kg/day for 6 weeks	Intraperitoneal injection	Reduce inflammation and oxidative damage. Improved acetylcholine levels in the brain	(Abbas et al. 2022)
Female Sprague Dawley rats injected with Aβ	20 mg/kg/day for 15 days	Intragastric intubation	Enhances neuronal viability. eliminate amyloid plaques and improve tau phosphorylation in Aβ1–42 infused rats	(Elibol et al. 2020)

#### Molecular mechanisms involved in the neuroprotective effects of thymoquinone

A molecular mechanism is a sequence of events involving molecules within a cell that culminate in a particular outcome or cell function. Numerous proteins, such as Nrf2, NF-κB, and TLR4, play vital roles in the prevention of brain diseases. Nrf2 is a transcription factor that is induced by oxidative stress, and Keap1 plays a crucial role in the regulation of ROS generation (Muchtaridi et al. 2024).

A recent review revealed that the primary mechanism underlying the various pharmacological characteristics of TQ is the enhancement of antioxidant status and the inhibition of inflammatory processes through the modulation of Nrf2 and NF-κB signaling through the PI3K/AKT pathway (Sadeghi et al. 2023). More recently, modulation of the Nrf2/HO-1 signaling pathway was reported to be the main mechanism responsible for the antioxidant effects of TQ through the blockade of Keap1, which modulates the Nrf2 signaling pathway and increases Nrf2 gene expression and nuclear translocation (Saadat et al. 2024). Furthermore, TQ controls oxidative stress and enhances Nrf2 expression in the pyramidal cell layer and the activity of HO-1, SOD, and CAT in the brains of obese rats (Mostafa et al. 2025). TQ help to preserve neuronal integrity and synaptic function, which are fundamental for maintaining cognitive capacity under metabolic stress through enhancing endogenous antioxidant defense in critical brain regions.

**Fig. 2:**
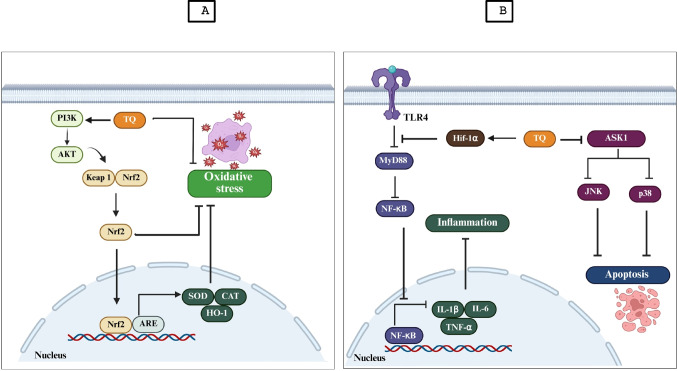
Molecular protective mechanisms of thymoquinone against oxidative stress, inflammation, and apoptosis. (**A**) TQ reduces oxidative stress by activating the PI3K/AKT/Nrf2 pathway and increasing the expression of antioxidant enzymes (SOD, CAT, HO-1). (**B**) TQ down-regulate neuroinflammation by blocking the TLR4/MyD88/NF-κB signaling pathway through Hif-1α activation, resulting in decreased expression of pro-inflammatory cytokines (TNF-α, IL-1β, and IL-6). Additionally, TQ has an antiapoptotic effect by inhibiting the ASK1/JNK/p38 signaling cascade, thereby preventing apoptosis. AKT: Protein kinase B; ARE: Antioxidant response element; ASK1: Apoptosis signal-regulating kinase 1; CAT: Catalase; Hif-1α: Hypoxia-inducible factor 1-alpha; HO-1: Heme oxygenase-1; IL-1β: Interleukin-1 beta; IL-6: Interleukin-6; JNK: c-Jun N-terminal kinase; Keap1: Kelch-like ECH-associated protein 1; MyD88: Myeloid differentiation primary response 88; NF-κB: Nuclear factor kappa B; Nrf2: Nuclear factor erythroid 2–related factor 2; p38: p38 mitogen-activated protein kinase; PI3K: Phosphoinositide 3-kinase; SOD: Superoxide dismutase; TLR4: Toll-like receptor 4; TNF-α: Tumor necrosis factor-alpha; TQ: Thymoquinone

NF-κB is an inducible transcription factor that regulates gene expression linked to inflammation, the innate immune response, apoptosis, and the stress response. In addition to regulating the inflammasome, NF-κB triggers the expression of several proinflammatory genes, such as those that encode cytokines and chemokines (Liu et al. 2017). Typically, Keap-1 is localized in the cytoplasm, but in response to high levels of oxidative stress, it does not complex with Keap-1, translocate into the nucleus and bind to antioxidant response elements, leading to the transcription of various cellular antioxidants (Muchtaridi et al. 2024). TLR4 is a transmembrane receptor that is essential for the innate immune response. TLR4 initiates intracellular signaling cascades via interactions with exogenous ligands at the surface of the cell membrane and with intracellular ligands (Kim et al. 2023). TQ exerts a neuroprotective effect on brain I/R injury via a mechanism involving inhibition of the TLR4/NF-κB signaling pathway by activating Hif-1α, regulating the polarization of M1/M2 microglia, and thus attenuating the inflammatory response of the brain (Zhao et al. 2024) (Fig. 2). Suppressing chronic neuroinflammation through these pathways is vital, as persistent inflammatory signaling directly impairs hippocampal neurogenesis and synaptic plasticity, processes indispensable for learning and memory functions.

Numerous inflammatory and hypoxic disorders are tightly linked to the TLR4 signaling pathway and Hif-1α. TLR4 promotes the release of proinflammatory mediators by activating the NF-κB pathway in B cells, and its upregulation in hypoxic microglia depends on Hif-1α. Thus, Hif-1α-mediated control of TLR4 expression in microglia could be a novel therapeutic target for a number of illnesses, including CNS hypoxia (Wei et al. 2023)**.** According to their findings, administering TQ following ischemic stroke considerably alleviated motor impairments and restored neurological function by lowering cerebral infarct volume, cerebral edema, and neuronal damage. The combination of TQ and HIF-hydroxylase inhibitor (DMOG) has neuroprotective effects, including improving motor dysfunction, reducing brain edema, and decreasing BBB extravasation. These effects are attributed to the downregulation of inflammation and upregulation of angiogenesis as well as neurogenesis through stimulation of the HIF-1α-VEGF/Nrf2-HO-1-TrkB-PI3K pathway (Amin et al. 2022). The initiation of neurogenesis and angiogenesis provides a direct route for neural repair and functional recovery, which underpins the restoration of cognitive and motor outcomes after brain injury.

TQ administered orally for 24 weeks significantly improved insulin signaling and glucose tolerance in mice fed a high-calorie diet. It also increased the protein expression of phosphorylated Akt (pAkt), a crucial part of the insulin signaling pathway, through SIRT-1/AMPKα-dependent signaling (Karandrea et al. 2017). Given that intact hippocampal insulin signaling (through pAkt) is essential for synaptic plasticity and long-term memory formation, this action provides a direct mechanistic link through which TQ could counteract HFD-induced cognitive decline. The activation of AMPK by TQ reduces obesity by inhibiting acetyl-CoA carboxylase 1, which is responsible for fatty acid synthesis (Ibrahim et al. 2024). Interestingly, the ability of TQ to ameliorate STZ-induced neurodegeneration was linked to the activation of the JNK protein, a mitogen-activated protein kinase (MAPK) that controls cell death, proliferation, and differentiation. By modulating neurodegeneration via the MAPK pathway via JNK1/2 and ERK1/2, TQ may affect STZ-induced neurodegeneration (Dalli et al. 2018).

According to earlier research, TQ may decrease neuronal damage by blocking apoptosis signal-regulating kinase 1 (ASK1), which activates the JNK and p38 MAPK pathways and is implicated in apoptosis and other cellular stress responses (Alrafiah 2021). ASK1 deficiency limits neurodegeneration (Kadowaki et al. 2005). These pathways control inflammatory cytokines such as TNF-α and oxidative stress, hence providing protection for brain function. Neuronal damage may result from the activation of ASK1, and TQ may inhibit apoptotic signals and protect neurons from injury by lowering ASK1. This anti-apoptotic effect is critical for preserving neuronal populations and connectivity within brain circuits which is responsible for cognitive processing.

Hypothalamic inflammation and glial fibrillary acidic protein (GFAP; a marker for astrocytic activation) overexpression in astrocytes are well described in obese animals, as are some cognitive and memory deficits (Bondan et al. 2019). The relationship between TQ and HFD-induced GFAP changes is not yet well-defined in the literatures, but TQ has shown potential to ameliorate neuroinflammatory processes by modulating signaling pathways, suggesting a possible protective role against HFD-induced neuroinflammation. TQ improved antioxidant activity and reduced the expression of GFAP and neuronal death in the hippocampal tissue of nonylphenol-treated rats. Moreover, an *in vitro* investigation using astrocytes isolated from the brains of mice revealed that TQ dramatically improved cell survival in the context of nonylphenol-induced cytotoxicity (Lotfi et al. 2022). Therefore, by mitigating astrocyte reactivity (GFAP), TQ targets a key driver of the neuroinflammatory milieu that disrupts synaptic efficiency and neurogenesis, thereby offering a pathway to protect against obesity-associated memory impairments.

#### Effect of thymoquinone on oxidative stress

An important factor in ischemic brain injury is inflammation mediated by ROS. Thus, TQ may decrease ischemic brain damage by activating the Nrf2/HO-1 pathway. According to a recent study, TQ is a potentially effective intervention therapy for cerebral ischemia because it modulates apoptotic and autophagic processes and reduces inflammation, oxidative stress, and neuronal cell death through the Nrf2/HO-1 pathway (Amin et al. 2021). The HIF-1α protein has been proposed as a putative transcription factor for stroke treatment because of its indirect effects on metabolism and cellular processes. According to recent research, TQ may function as an HIF-1α activator to regulate autophagy, reduce the volume of the infarct following the commencement of ischemic stroke, and reduce cell death. Accordingly, TQ may have a neuroprotective effect by reducing inflammation, promoting angiogenesis, and stimulating neurogenesis through the activation of HIF-1α-VEGF/Nrf2-HO-1-TrkB-PI3K (Amin et al. 2022). The combined effect of reducing neuronal loss during promoting new neuron synthesis (neurogenesis) and vascular support (angiogenesis) directly targets the structural and functional substrates vital for cognitive recovery and resilience. On the basis of this information, it seems that TQ has the ability to mitigate the brain and improve cognitive function in response to various toxins through antioxidant and anti-inflammatory mechanisms.

#### Effects of thymoquinone on obesity and weight gain

Recently, supplementation with TQ essential oils was reported to decrease final body weight, weight gain, blood glucose levels and insulin resistance (HOMA-IR). This effect was associated with an improved lipid profile and antioxidant system (Alanazi et al. 2023). Similarly, in rats, high-fructose diet-induced metabolic syndrome is prevented by TQ, which may be mediated by the PPAR pathway (Prabhakar et al. 2015). It is debatable whether *Nigella sativa* reduces body weight and the risk of obesity. However, body weight and other anthropometric indices can be significantly decreased with long-term *Nigella sativa* supplementation (6–12 weeks). Because *Nigella sativa* oil contains more fatty acids and TQ than does *Nigella sativa* powder, it is more effective than the powder for lowering body weight. The suppression of apatite, reduction in caloric intake, and blockage of intestinal glucose absorption are potential mechanisms by which *Nigella sativa* decreases body weight (Al Asoom 2022).

**Fig. 3:**
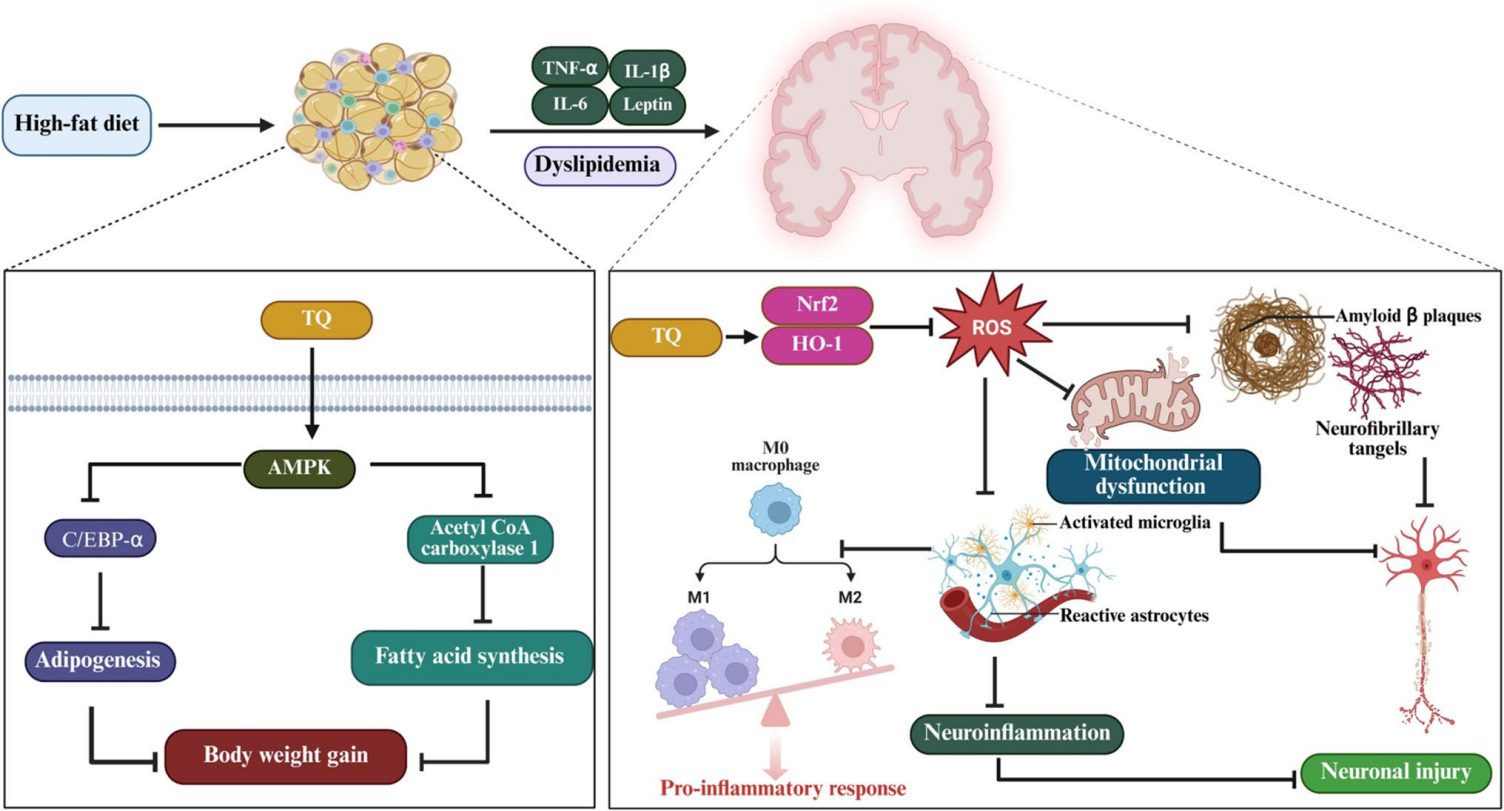
Neuroprotective pathways of thymoquinone against High-Fat Diet-induced obesity and neuronal Injury. In neuronal cells, TQ activates the Nrf2/HO-1 antioxidant pathway, resulting in decreased reactive oxygen species production and improved mitochondrial function. Through attenuation of oxidative stress, TQ suppresses microglial activation, reactive astrocyte formation, and pro-inflammatory macrophage polarization, thereby reducing neuroinflammation. Additionally, TQ limits the formation of amyloid-β plaques and neurofibrillary tangles, ultimately protecting against neuronal injury. In adipocytes, TQ mitigates body weight gain by activating AMPK, leading to decreased fatty acid production and adipogenesis through the inhibition of acetyl-CoA carboxylase 1 and C/EBP-α. AMPK: AMP-activated protein kinase; C/EBP-α: CCAAT/enhancer-binding protein alpha; HO-1: heme oxygenase-1; M1/M2: macrophage phenotypes; Nrf2: nuclear factor erythroid 2-related factor 2; ROS: reactive oxygen species; TQ: thymoquinone

An atypical antipsychotic medication for psychotic illnesses is olanzapine (OLZ). Research has linked OLZ to adverse metabolic effects such as insulin resistance, obesity, and hypertension. TQ attenuated the adverse effects of OLZ and decreased body weight, food intake, and lipid peroxidation and increased GSH levels. TQ also reduces glucose, serum lipid, and leptin levels (Kaviani et al. 2023). According to this study, TQ is useful in reducing metabolic anomalies because of its antioxidant action, which also helps to regulate lipid metabolism and glucose homeostasis, hence improving brain plasticity in obese/overweight individuals. Similarly, compared with HFD-fed rats, TQ-fed rats may have increased antioxidant levels and lower levels of inflammatory and oxidative stress mediators. Neuronal damage is decreased with TQ therapy. Thus, TQ is a potentially effective treatment for obesity-related alterations in the morphology of neurons in the cerebellum (Alrafiah 2021). Rats were used in a recent study to assess the antiobesity and antihyperglycemic effects of TQ. TQ reduces obesity and diabetes indices by regulating crucial adipokines, essential lipid-metabolizing enzymes, and AMPK/p-AMPK in HFD-induced obese mice (Ramineedu et al. 2024) and suppressing brain damage (Mostafa et al. 2025) (Fig. 3).

#### Translational perspectives and future directions

The preclinical literature in this review highlights TQ’s multimodal potential to counteract the pathological cascade linking obesity to cognitive decline. However, to translate these promising findings into clinical application presents several challenges which must be addressed. To be more concise, dosage optimization remains a complex hurdle. As, effective doses and treatment durations in rodent models vary significantly (e.g., (Karandrea et al. 2017). Translating these regimens to humans requires careful pharmacokinetic studies to establish bioavailability and blood–brain barrier movement, in addition to, optimal dosing schedule for usage. Moreover, data on long-term safety in human are insufficient till now. While animal studies report good tolerability, comprehensive toxicological profiles are necessary to define a clear safety profile.

The most critical gap is the lack of direct clinical evidence for cognitive benefits in obese individuals. Although clinical trials demonstrate that Nigella sativa supplementation can improve metabolic parameters (Alanazi et al. 2023; Al Asoom 2022), no study to date has investigated its direct impact on cognitive performance, brain imaging biomarkers, or the incidence of cognitive decline in overweight or obese human. Future research must prioritize well-designed randomized controlled trials which link metabolic endpoints (e.g., insulin sensitivity, inflammatory markers) with validated neuropsychological and cognitive assessments and advanced neuroimaging to bridge this vital gap. Investigating TQ as an adjunct therapy to lifestyle interventions and defining its potential synergies with other neuroprotective agents will also be crucial steps forward. Interestingly, TQ’s core mechanisms involve modulating oxidative stress and inflammation which are shared by other promising classes of natural compounds. For instance, mechanistic insights highlight the neuroprotective potential of acyclic sesquiterpenes like nerolidol and farnesol, as well as flavonoids such as hispidulin and diosmin, through pathways analogous to those activated by TQ (Singh and Singh 2025; Singh et al. 2025). This highlights a wide therapeutic framework for future research, where comparative efficacy and synergistic combinations across phytochemical classes could prove to be highly valuable.

According to this review, TQ might be potentially useful for treating obesity and its neurological consequences. TQ might be used in combination with other antioxidants or drugs. The collective mechanistic evidence suggests that, TQ targets the core pathological triad that drives obesity-related brain dysfunction through, ameliorating systemic metabolism, quenching central oxidative stress, and dampening neuroinflammation. Future research is necessary to confirm the effectiveness of TQ in ameliorating obesity and its neurocognitive consequences in obese/overweight individuals.

#### Current status and prospects of clinical application of thymoquinone

Currently, TQ is mainly in the preclinical research stage for the majority of diseases; however, a limited number of early-stage human clinical trials have explored its use as an adjunctive therapy for conditions such as diabetes, epilepsy, and periodontitis. To date, there have been no significant clinical advancements or FDA approvals for TQ as a pharmaceutical drug so far. Summary of clinical trials of TQ and quality assessment has been reviewed recently, pointing that further well-designed, large-scale clinical studies are warranted to establish the efficacy, optimal dosing strategies, and long-term safety of TQ in human populations (Gouda et al. 2025).

## Conclusion

Thymoquinone (TQ) has shown potent neuroprotective potential in preclinical studies. As, thymoquinone (TQ) mitigated overweight and obesity and their associated neurological damage possibly via improving metabolic function. In addition to the improvement of body weight gain and metabolic status in obese rats. It is shown to augment antioxidant enzymes, maintain redox equilibrium, and diminish neuroinflammation and apoptosis via multiple signaling pathways to achieve neuroprotection and maintain brain health. TLR4, Nrf2, NF-κB, and MAPK are key proteins involved in the signaling pathways through which TQ may help prevent brain disorders. The therapeutic usefulness of these proteins in a variety of brain disorders remains challenging, although they represent novel treatment approaches. However, translating this into effective human therapies, especially for conditions such as obesity-related brain dysfunction, has significant translational hurdles. The foremost challenge is that TQ's possess poor pharmacokinetic profile, characterized by low aqueous solubility, rapid metabolism, and systemic clearance, resulting in low oral bioavailability and insufficient delivery to target tissues. his limitation is especially pertinent in overweight/obese populations, where altered drug distribution and metabolic states may further compromise efficacy. To overcome these obstacles, advanced formulation strategies are essential. Research into nano-formulations such as liposomes, polymeric nanoparticles, lipid-based delivery systems, and solubility enhancers has shown promising results in improving TQ's stability, bioavailability, and brain uptake in animal models. Future research must be directed towards pharmacokinetic optimization and robust clinical trials employing these advanced formulations as well. Establishing these precise delivery strategies is essential for harnessing TQ's full potential in the prevention and treatment of brain dysfunction.

## Data Availability

All data supporting the findings of this review are included within the article.
